# Mortality in survivors of adolescent and young adult cancer: progress and opportunities

**DOI:** 10.1016/S2468-2667(24)00277-9

**Published:** 2025-01

**Authors:** Michael E Roth, Amy M Berkman

**Affiliations:** Division of Pediatrics, The University of Texas MD Anderson Cancer Center, Houston, TX 77030, USA (MER); Department of Pediatrics, Duke University School of Medicine, Durham, NC, USA (AMB)

Survivors of adolescent and young adult (AYA) cancer (ie, those aged 15 to 39 years at diagnosis) are a large and growing population. There are more than 2 million survivors of AYA cancer in the USA alone,^1^ and efforts targeted at reducing late morbidity and mortality are urgently needed. In *The Lancet Public Health*, Taylor Hughes and colleagues^2^ evaluate the risk of mortality after a diagnosis of cancer as an AYA over a median of 9 years from diagnosis. Including 24 459 individuals with primary AYA cancer in the Alberta AYA Cancer Survivor Study, they found that all-cause cumulative morality was 35·6% (95% CI 34·6–36·7) at 30 years from diagnosis. This translated to an 11-fold increased risk of mortality in the overall cohort and a 4-fold increased risk of mortality among those who reached 5 years of survival compared with expected mortality in the general population. Cancer recurrence or progression was the main driver of excess mortality, whereas beyond 10-years from diagnosis, subsequent primary neoplasms (SPN) and non-cancer causes of death became more prevalent. Encouragingly, the authors found reductions in mortality for AYAs diagnosed with cancer in more recent decades, largely the result of decreased deaths due to cancer recurrence or progression. These results are similar to those of previous population-based survival analyses using data from the Surveillance, Epidemiology, and End Results database and California Cancer Registry, which found improvements in primary cancer mortality over time, as well as persistently elevated long-term risks of non-cancer and SPN mortality among survivors of AYA cancer.^3–5^

When evaluating mortality by cancer type, Hughes and colleagues found that survivors of endometrial cancer, testicular cancer, and Hodgkin lymphoma had the highest burden of deaths due to cause other than cancer recurrence or progression, which was particularly evident beyond 10 years from diagnosis. These results are consistent with findings from a previous California Cancer Registry study of 5-year survivors of AYA cancer (n=162 317), which showed that survivors of Hodgkin lymphoma, cervical or uterine, and testicular cancer had the highest burden of non-cancer cumulative mortality at 30-years of follow-up.^5^ By confirming these findings, the authors further draw attention to the importance of survivorship-focused care, particularly for groups with known treatment-related risks. Among survivors of Hodgkin lymphoma, cumulative mortality due to SPNs was 8·0% and due to circulatory diseases was 4·4%,^2^ both of which are well known risks in survivors of this cancer type.^6^ However, findings showing the burden of deaths due to circulatory diseases, endocrine disorders, and SPNs among survivors of AYA endometrial cancer, and deaths due to SPNs and external causes among survivors of testicular cancer are more novel and warrant further investigation.

Despite meaningful improvements in 5-year survival for AYAs diagnosed with cancer, there are many opportunities to further improve short-term and long-term outcomes. As Hughes and colleagues noted,^2^ survival for AYAs with some prevalent AYA cancers, such as sarcomas and CNS tumours, has been poor and stagnant for many decades. The recent success of collaborative AYA studies within the National Cancer Institute Clinical Trials Network (NCTN) highlight an opportunity to more efficiently improve outcomes for AYAs diagnosed with cancer. For example, the SWOG S1826 study (NCT06094556) evaluated the addition of nivolumab or brentuximab to standard chemotherapy for patients with advanced Hodgkin lymphoma and completed accrual 1 year earlier than anticipated. The study recently reported that patients randomly allocated to receive nivolumab had significantly improved progression-free survival and, notably, very few patients received radiation.^7^ Consequently, in addition to improved short-term survival, the reduction in use of radiation is expected to lower the risk of late effects, thereby decreasing long-term morbidity and mortality. The NCTN AYA trial portfolio continues to expand, and studies targeting AYA cancers with the poorest prognoses and those with the highest risk for severe and life-threatening late effects should be prioritised.

There is an urgent need to expand patient access to comprehensive survivorship care, prioritising the screening and management of late effects and subsequent malignancies. Special attention should be focused on addressing the needs of survivors from minoritised populations and those with few resources and limited access to care.^8^ Although the current study could not evaluate associations with race and ethnicity due to data limitations in the Alberta registry, previous research has highlighted racial and ethnic disparities in early and late mortality among survivors of AYA cancer.^9,10^ Additionally, longer follow-up, especially for survivors treated within the past decade, is essential to better understand the long-term risks associated with novel agents, as the late effects of these treatments remain largely unknown. It is uncertain whether the observed reductions in late mortality will persist as these survivors live longer post-diagnosis.

This study adds to previous research by further delineating the excess risk of mortality faced by survivors of AYA cancer. As the population of AYA cancer survivors continues to grow, additional efforts are needed to further increase both short-term and long-term survival. Priorities should include improving access to early diagnosis, high-quality cancer care, and clinical trials, as well as developing interventions to reduce the risks of late mortality.
